# Individual geographic mobility in a Viking-Age emporium—Burial practices and strontium isotope analyses of Ribe’s earliest inhabitants

**DOI:** 10.1371/journal.pone.0237850

**Published:** 2020-08-27

**Authors:** Sarah Croix, Karin Margarita Frei, Søren Michael Sindbæk, Morten Søvsø

**Affiliations:** 1 Centre for Urban Network Evolutions (UrbNet), School of Culture and Society, Aarhus University, Højbjerg, Denmark; 2 National Museum of Denmark, Copenhagen, Denmark; 3 Museum of Southwest Jutland, Ribe, Denmark; University at Buffalo - The State University of New York, UNITED STATES

## Abstract

Individual geographic mobility is a key social dynamic of early Viking-Age urbanization in Scandinavia. We present the first comprehensive geographic mobility study of Scandinavia’s earliest emporium, Ribe, which emerged around AD 700 in the North Sea region of Denmark. This article presents the results of strontium isotope analyses of 21 individuals buried at Ribe, combined with an in-depth study of the varied cultural affinities reflected by the burial practices. In order to investigate geographic mobility in early life/childhood, we sampled multiple teeth and/or petrous bone of individuals, which yielded a total of 43 strontium isotope analyses. Most individuals yielded strontium isotope values that fell within a relatively narrow range, between ^87^Sr/^86^Sr = 0.709 to 0.711. Only two individuals yielded values >^87^Sr/^86^Sr = 0.711. This suggests that most of these individuals had local origins but some had cultural affinities beyond present-day Denmark. Our results raise new questions concerning our understanding of the social and cultural dynamics behind the urbanization of Scandinavia.

## Introduction

Recently, research on human migration in the past has seen a revival in archaeology, largely owing to the development of methods such as aDNA and strontium isotope analyses [e.g. 1], and is highly relevant in the context of the urbanization of Scandinavia in the early Viking Age. From the 8^th^ century onwards, new settlement sites that functioned as nodes for maritime trade emerged along the coasts of the North and Baltic Seas, for example, in Ribe, Kaupang, Birka, Hedeby, Reric/Gross Strömkendorf, and Åhus [2, 3]. Through their own travels and/or interactions with visitors (merchants, missionaries, slaves), the inhabitants of these urban sites, or “emporia,” had extensive regional and supra-regional contacts. In previous studies, several approaches, including comparative analyses of burial practices, contextual analyses of material culture in domestic contexts, textual analyses of travel accounts, and ancient DNA and isotope studies, have been applied, in order to identify local and foreign/non-local individuals in the emporia of Northern Europe [4–14]. However, combining these approaches to yield convincing interpretations of the emporia’s social and cultural composition is a complex interdisciplinary exercise.

**Fig 1:**
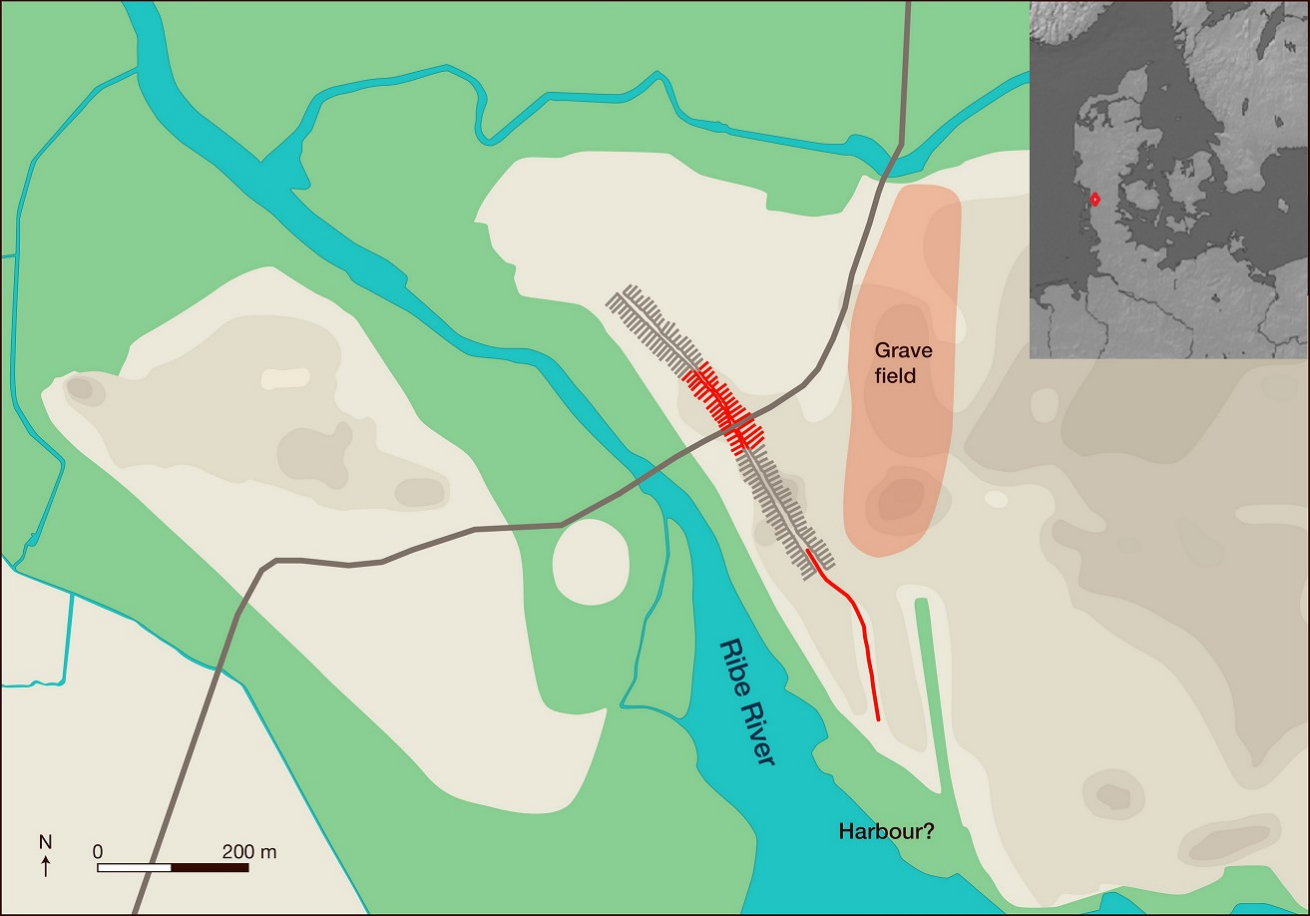
Ribe in the early Viking Age.

Ribe was the earliest major emporium in Scandinavia, dating to around AD 700, when a trading center with far-reaching contacts emerged on the northern bank of the River Ribe [15] (Fig 1). Contemporary with the early town was a burial ground with several forms of burial, including those of men, women, and children [16, 17]. This cemetery and its cultural landscape provided the source material for this article, which engages in an interdisciplinary discussion of cultural affinity and geographical origin, based on a comparison of strontium isotope analyses of multiple samples from 21 individuals, and a study of burial practices. The results provide new insights into Ribe’s early urban community, and hint at the complex social and cultural processes linked to human geographic mobility in the context of the urbanization of Scandinavia.

Ribe is located near the coast of Southwest Jutland, in present-day Denmark, facing the Wadden Sea. The map presents a reconstruction of the site in the early Viking Age and of the natural topography: dry areas are depicted in grey shades, and have elevations between 2 and 6 meters above sea-level, whereas wet areas, with elevations under 2 meters above sea-level, are depicted in green. A number of excavations on the north side of the River Ribe have allowed a reconstruction of Ribe’s townscape in the 8^th^ and 9^th^ centuries. The main settlement area consisted of narrow, rectangular building plots, represented here by the plot boundaries. Some have been documented archaeologically (red lines), others reconstructed (light grey lines). The plots were placed in two rows on either side of a street running in a NW-SE direction. A main road (thick grey line) connected the site to inland areas. The surface of the burial ground to the east has been estimated to cover c. 8–9 ha. Graphics: Morten Søvsø and the Graphics Department, Moesgård Museum. Background map retrieved from Natural Earth. Free vector and raster map data @ naturalearthdata.com. Reproduced under the copyright terms of use of Natural Earth.

## Background: Viking-Age towns as meeting places

Addressing the question of cultural affinity and geographical origin is of paramount importance for understanding social dynamics in the early Scandinavian towns. The emergence of these more or less permanent coastal settlements, whose economy focused on trade and crafts, is considered one of the most important societal changes in the essentially agrarian and pastoral communities of Northern Europe in the late Iron Age/early Middle Ages [18–21]. Although there is much debate about what (and who) prompted the urbanization of Scandinavia, especially concerning its ties to royal power [22–24], one fact remains: Few people were already settled in the locations where the key Viking-Age sites of Birka (present-day Sweden), Kaupang (present-day Norway), Hedeby (present-day Germany) and Ribe (present-day Denmark) appeared and thrived from the early 8^th^ to the late 10^th^ centuries, but at one point or another, all these sites hosted a large, permanent population. Their success implies that some people moved there, and stayed. Furthermore, they acted as nodal points in a long-distance exchange network [25], and visitors from near and far would have been common, as attested by both contemporary textual accounts and artefactual finds [4–8]. The history of populating the Viking-Age towns involves geographic mobility, and is marked by encounters between individuals with cultural ties and geographic origin in the towns’ immediate hinterland, but also beyond.

In the context of Scandinavia’s early towns, questions of origin have often been treated based on burial remains. From an archaeological perspective, burial practices, defined by a number of criteria—such as the treatment of the body, grave markers, and the objects found with the remains of the dead—have been used to discuss cultural affinity, which in some cases may be related to specific regions. This approach, which has been widely used and debated for decades in early medieval archaeology [e.g. 26, 27], has also been applied in an increasingly nuanced way to the interpretation of burial practices attested at the Scandinavian emporia—from reflecting the appurtenance to an ethnic group, to the expression of a hybrid identity [e.g. 11–12]. In recent decades, strontium isotope analyses have been added to the archaeologists’ toolkit, shedding light on research questions related to the possible geographic mobility of individuals and their place of origins [e.g. 28, 29]. In particular, the population of Birka has been the subject of several case studies that applied the strontium isotope methodology to a number of skeletal remains, which revealed the geographic mobility of some of the individuals buried there [13, 14]. Combining the investigations of cultural affinity revealed by burial practices with that concerning geographical origin indicated by the results of strontium isotope analyses of the human remains may in some cases expose discrepancies, which can be interpreted as reflecting complex social dynamics, negotiations of identity, and cultural processes of hybridization, adoption, and/or rejection.

Besides interpretative challenges, such interdisciplinary studies use a different vocabulary to convey the notion of geographic origin. Within the field of isotopic studies, this notion is typically referred to in terms of “local” and “non-local”. The word, in this context, refers to locality, which may be of considerably variable extent, rather than to a small region defined by its relation to a specific place. In artefact-, burial-, and settlement studies, archaeologists may associate cultural traits to a place or type of places and to regions, which scale may vary, depending on the research question, area of study, and time period involved. In the case of the Scandinavian emporia, it may signify a few kilometers’ range, sometimes designated as the “local” area, or Scandinavia as a whole, for example in contrast to the rest of Europe [e.g. 9, 10]. The interdisciplinary endeavor attempted here thus requires clarity in the intended geographic designation.

## Ribe’s emporium and cemetery: An overview

Since its archaeological discovery in the 1970s, Ribe’s emporium has occupied a special place in the study of early medieval urbanism and in Viking-Age archaeology in particular [15, 30, 31]. The emporium itself consists of a dense settlement area dated to the 8^th^–10^th^ centuries, where the abundant artefactual material leaves no doubt as to its special functions: trade -local, regional, and long-distance- and specialized manufacture of crafted goods. Remains of houses also indicate a resident population [32]. Traces of an additional, possibly agrarian settlement that existed partly contemporaneously with the emporium (8^th^-11^th^ centuries) have been found in the immediate vicinity, but their extent and character are less clear. On the south side of the River Ribe, a Christian cemetery, founded c. 860, was excavated in 2008–2009 and 2011–2012 [33].

During the early Viking Age, Ribe’s trade network grew to encompass Norway and the Baltic Sea area, and eventually, to form distant links to the Mediterranean and the Middle East [34, 35]. However, the bulk of Ribe’s attested contacts points to the lower North Sea region and the Rhine river valley, whence industrial products of the eastern Frankish kingdom, such as glass, pottery, and quern stones, traveled to the emporium [36, 37].

**Fig 2:**
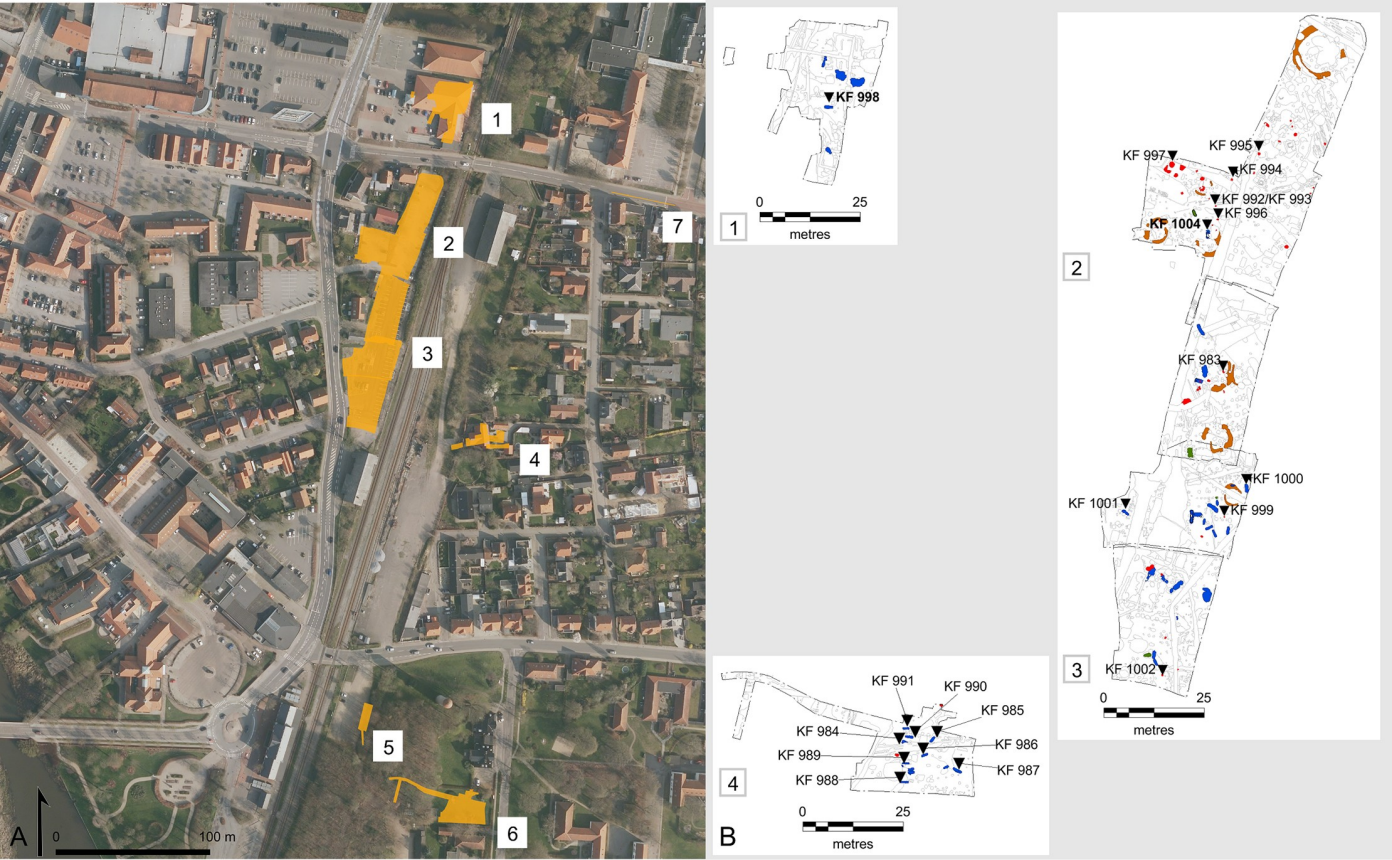
Excavated areas at Ribe’s earliest cemetery and spatial distribution of burials by burial type. (A) The site of Ribe’s earliest cemetery has been occupied nearly continuously since the Viking Age. The aerial photograph shows the location of the excavated areas of the cemetery in Ribe’s urban landscape today (yellow polygons): 1) SJM 129 Seminarievej, 2012; 2) ASR 8 Rosen Allé, 1989; 3) SJM 348 Rosen Allé, 2014–2016; 4) ASR 937 DSB Øst/Dr. Dagmars Vej 5a, 1990, 1991, 1998; 5) 43M70 Skt Nicolai kirkegård, 1970; 6) ASR 1000 Ribelund II, 1991–1992; 7) ASR 1030 Seminarievej, 1992. (B) A large number of features from different periods, dated to the Viking Age up to modern times, have been excavated at the site. Most burial features belonging to Ribe’s earliest cemetery were found at 1) SJM 129 Seminarievej, 2012; 2) ASR 8 Rosen Allé, 1989; 3) SJM 348 Rosen Allé, 2014–2016; and 4) ASR 1000 Ribelund II, 1991–1992. All features from these excavations are shown on detail plans (light grey lines), and the burial features are marked according to their burial type (see Table 1): inhumations (blue), cremation (red), burial features with non-human remains (green), and ring ditches (orange). The position of the burials with preserved human remains submitted to strontium isotope analyses is indicated by black down-pointing triangles and their corresponding laboratory number. The two non-local individuals identified by strontium isotope analyses are indicated in bold. Graphics: Sarah Croix. Contains data from GeoDanmark, Styrelsen for dataforsyning og effektivisering (Agency for Data supply and Efficiency) and Danske kommuner, “GeoDanmark Ortofoto”, retrieved July 2020. Reproduced under the copyright terms of use of GeoDanmark.

The earliest documented cemetery in Ribe is located to the east of the emporium, on the north side of the River Ribe. Graves have been found in this area on several occasions, and there have been two main excavation campaigns, in 1988–1989 and 2014–2016. To date, exactly 100 archaeological features have been interpreted as belonging to the burial ground, including a number of grave markings (ring ditches), animal offerings, and animal burials (Table 1; Fig 2). Based on a series of ^14^C dates, artefact chronology, and stratigraphy, the site has been dated to the 8^th^ century and early 9^th^ century, with a small cluster of graves in the southern part of the site dated to the 9^th^ and 10^th^ centuries [38]. This division is not absolute, however, as several graves could not be dated, but are considered to belong to the same ensemble, owing to similar degrees of preservation and the consistency of the burial practices with dated graves. Furthermore, the area has seen nearly continuous use from the Viking Age until today, which has resulted in the truncation of many features. The common practice of placing cremation deposits on the surface or in shallow pits, which are easily destroyed, means that the original number of Viking-Age graves is unknown, and almost certainly underestimated. The current distribution of the graves shows that a large area of at least 8–9 ha was used as cemetery. There are several instances of intercutting, and together, these factors suggest that originally there may have been at least several hundred graves. In all likelihood, the cemetery belonged to the inhabitants of the emporium, and possibly visitors who died during their stay and the inhabitants of the agrarian settlement in the immediate vicinity as well.

**Table 1:** Overview of burial features documented at Ribe’s earliest cemetery.

		Nb of burial features	Nb of burial features with preserved bone material submitted to osteological analysis	Nb of human individuals based on osteological analysis	Nb of burial features with grave goods	
**Inhumation: 44**						
• 28 certain						
• 16 possible						
	Earth-burials	35	21	20	7	
		13 graves W-E +/-		20 single graves		
		22 graves S-N +/-		1 grave with indeterminate mammal remains		
	Inhumation in coffins, W-E +/-	5	2	2	1	
		2 with rectangular coffin (including 1 double)		2 single graves		
		2 with log/trough				
		1 with chest				
	Inhumation in coffins, S-N +/-	4	0	0	1	
		3 with logboats				
		1 with log/trough				
**Cremation: 45**						
• 41 certain						
• 4 possible						
	Pit deposits	32	31	33	15	
				29 single graves		
				2 double graves		
	Urn deposits	6	6	6	4	
				4 single graves		
				1 double grave		
				1 grave with indeterminate mammal remains		
	Surface deposits	3	2	1	2	
				1 single grave		
				1 with indeterminate remains		
	Disturbed / possible cremations	4	2	2	4	
				Context uncertain		
**Burial features with non-human remains: 4**						
	Animal burials	2	2	0	1	
				1 double grave (horse, dog)		
				1 single grave (dog)		
	Special deposits	2	2	0	0	
				1 single horse deposit		
				1 deposit with multiple animal remains		
**Ring ditches: 7**						
		3 with cremation remains	3	3	0	
		4 without		1 single grave		
				2 context uncertain		

100 archaeological features excavated on the north side of the Ribe River have been interpreted as belonging to Ribe’s earliest cemetery. The table presents a general overview of the data divided by main burial types. The number of burials with preserved bone material submitted to osteological analysis is indicated for each burial type, as well as the number of human individuals identified by osteological analysis. These figures do not include the cases where the outline of the body was recorded as a darker coloration of the sediments at the bottom of the grave pit, although these do represent human individuals as well. The Ribe graves were generally poorly equipped in terms of grave goods. The number of burials containing objects is indicated for each burial type.

The human and skeletal remains from Ribe’s earliest burial ground are publicly deposited and accessible by others in a permanent repository at the Museum of Southwest Jutland, Ribe, Denmark, on record under site numbers 43M70, ASR 8, ASR 937, ASR 1000, ASR 1030, SJM 129, and SJM 348. According to Danish law, the Museum of Southwest Jutland has full ownership and responsibility of the archaeological remains and granted all necessary permits for the studies described in this paper, following all relevant regulations.

All in all, human and animal skeletal remains were preserved in 71 of the 100 certain and probable burial features documented at the cemetery. All were submitted to osteological analyses, which were conducted by Susanne Østergaard, Department of Conservation and Natural Science, Moesgård Museum [39]. Owing to poor preservation condition of unburnt bone, skeletal remains were not preserved in all inhumation graves. In some cases, the outline of the skeleton could be documented as a darker coloration of the sandy sediments at the bottom of the grave pit, allowing adding them to the total count of certain graves (28 ex.). In 16 cases, however, no skeletal remains could be associated to the burial features with certainty during excavation, and the interpretation of these features as possible inhumation graves is based on similarities in form, dimensions, depth, fill, and deposited artefact types with well-attested graves. The preservation conditions were more favorable to burnt bone material, and their unambiguous association to 41 archaeological features allows identifying them as certain cremation graves. However, in two cases burnt bone remains described in the field were not kept and therefore could not be included in the osteological analysis; the osteological analysis was inconclusive in identifying the burnt bone remains as belonging to humans in two further cases; and the degree of disturbance of two archaeological features in which human bone remains were found implies that these may only be considered as possible cremation graves.

Unburnt human and animal remains from 27 burial features were examined, as well as burnt human and animal remains from 44 burial features. The analysis identified 67 human individuals, 57 of which from single graves and 6 from double graves. The remaining 4 human individuals identified were associated to disturbed contexts, which does not allow deciding on whether they belonged to single or double graves. The heterogeneity of the material means that different analytical methods were applied for sex and age estimation.

For sex estimation, sex-specific traits of the cranium and the os coxae, as well as the skeleton’s general morphology, were assessed, when possible [40–42]. Owing to the extensive fragmentation of the cremated material, a number of other, less reliable methods were used, such as the measurement of the larger long bones’ thickness, and dental morphometrics [43]. Although these analyses aimed to assess as many traits as possible, sex estimation is in most cases only putative. It should be noted that the osteological analyses were performed without prior knowledge of the artefacts deposited in the graves, with the aim of producing independent results, which could then be compared to the archaeological sex estimation at a later stage of interpretation.

Age estimation was conducted based on knowledge of skeletal development from birth to senescence, including dental eruption/wear, timing of bone fusion, signs of ageing in articulations, especially between vertebrae and the iliofemoral joint [42]. As not all parts of the skeletons were represented, owing to preservation and/or cultural processes (e.g. selection of remains from the pyre in the case of cremation), it was aimed at combining as many methods as possible, adapted to each individual case. As a result, age estimates were generally expressed in broad age categories, and for 19 individuals it was not possible to narrow their age at death further down than to adult years.

## Analyses of mortuary practices

Besides being at the crossroads of long-distance exchange routes, Ribe is also located in a complex cultural landscape. On the one hand, its position in Jutland and north of the Danevirke, the historical border of the Danish kingdom, positions Ribe in a Southern Scandinavian cultural sphere; on the other hand, it is located in a marginal position in present-day Denmark, looking towards the Wadden Sea, and in relatively close proximity to other cultural groups, in Frisia and Saxony, and to the North Sea region as a whole. On several levels, the cultural landscape of Southwest Jutland seems to have shared many similarities with that of the Northern and Eastern Frisian regions [44, 45]. Burial practices at Ribe and at Ribe’s surrounding sites differ indeed from those attested for the rest of Jutland [17]. Ribe’s burial ground stands out by a higher degree of diversity in burial practices.

**Fig 3:**
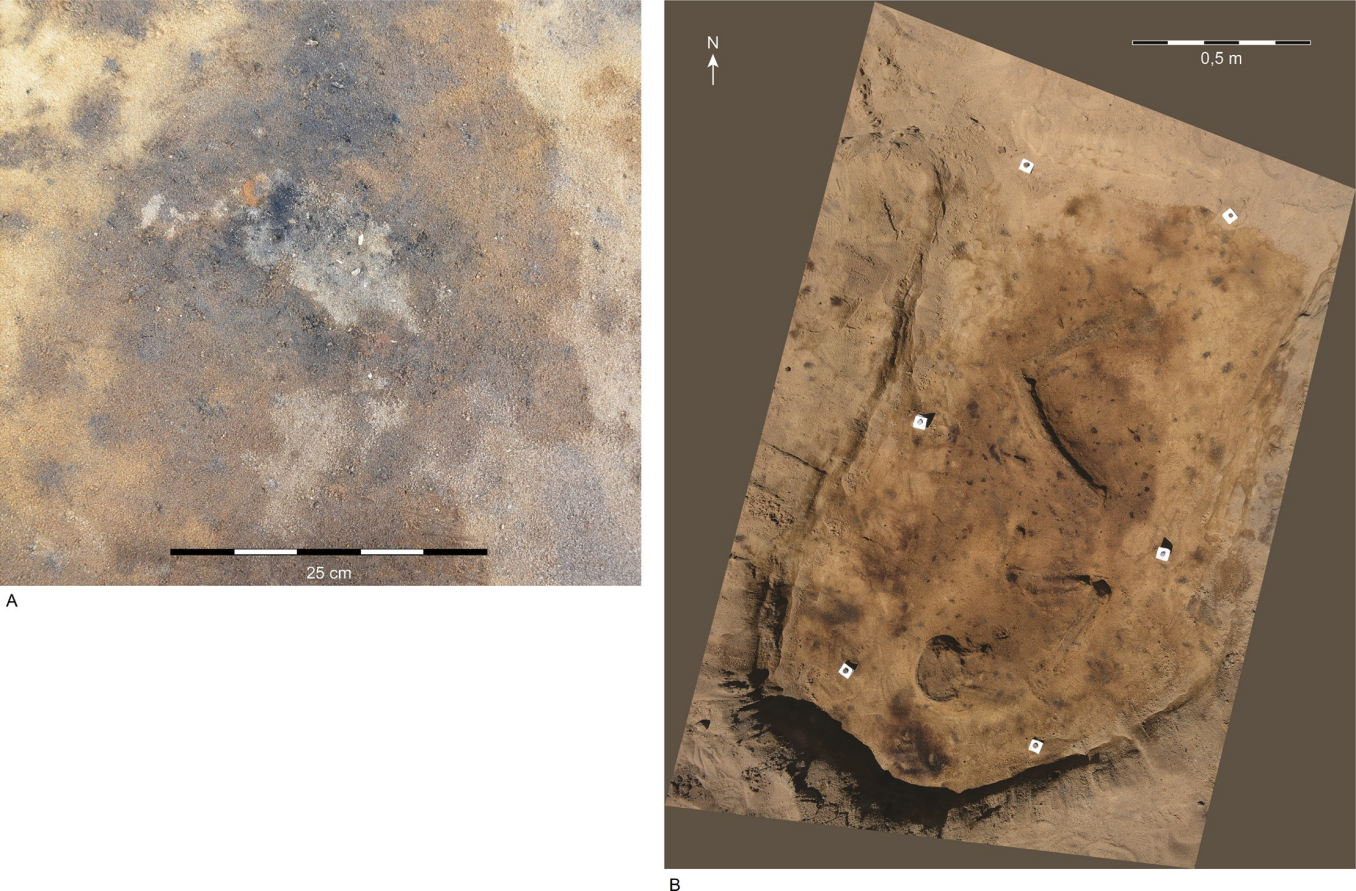
Cremation deposits and inhumations at Ribe’s earliest cemetery.

This diversity is evident in the highly varied treatment of the dead, starting with the various modes of treatment of the corpse, such as inhumation and cremation (Fig 3). In the case of inhumation, graves display variations in the orientation of the grave pit, the orientation of the body, the absence/presence of containers for the body, and their type, the absence/presence of artefacts, and their type, the absence/presence of animal remains, and their species. For the cremations, the generally circular, small form of the “grave,” that is, the pit receiving the pyre debris, does not display any pattern in orientation. However, the absence/presence of containers, and their type, may be assessed, as may the absence/presence of artefacts and animals.

Various burial practices were carried out at Ribe’s earliest cemetery, the most common consisting of cremated human, animal, and artefactual remains retrieved from the pyre and deposited indiscriminately in a shallow pit (A: SJM 348, G42/K850), and of simple inhumations in oblong pits (B: SJM 348, G19/K263). Photos: Aarhus University/Museum of Southwest Jutland.

When taking all the foregoing factors into account, the degree of variation in burial practices is striking, especially for the early phase of the cemetery, where inhumation graves were mostly oriented W–E or S–N; most did not involve any container, but there are several examples of coffins or logboats. The dead could be placed in a supine position, especially for W–E graves, whereas they were mostly laid in a crouched position in S–N graves. Inhumation graves rarely contained any deposited objects or animals, apart from a few instances of dress fittings, ornaments, coins, and single horse teeth. Cremation graves may be divided into three main types: one where the pyre remains were deposited in a small pit, one where the cremated human remains were carefully retrieved from the pyre and redeposited in an urn, and one where pyre remains seem to have been left as small deposits on the surface. Both pottery of regional type and Frankish imports could be used as urns. For both deposits in pits and in urns, the human remains were often mixed with the cremated remains of animals and various objects, such as small personal implements, tools, riding equipment, jewelry, and so on. Animals include various domestic mammals, various species of birds, and even fish in one case. Animals sometimes also received their own burials, as shown by one horse inhumation with harness and saddle, and one dog inhumation.

Theoretically, these variations may result from differences of treatment due to the social status, age at death, and/or gender of the individuals. However, variations in the degree of expenditure involved in the burial process (e.g. amount and quality of grave goods, use of logboat as coffin) cannot be considered to be reliable indicators of social status in this context, as each burial sub-type comprises a small number of relatively more lavish cases. Also, children, adults, and elderly are all represented among the different burial types, including male and female individuals, when sex could be provisionally estimated. Therefore, multiple cultural affinities should be considered the most likely explanation for the observed diversity, rather than age at death and gender identity.

**Fig 4:**
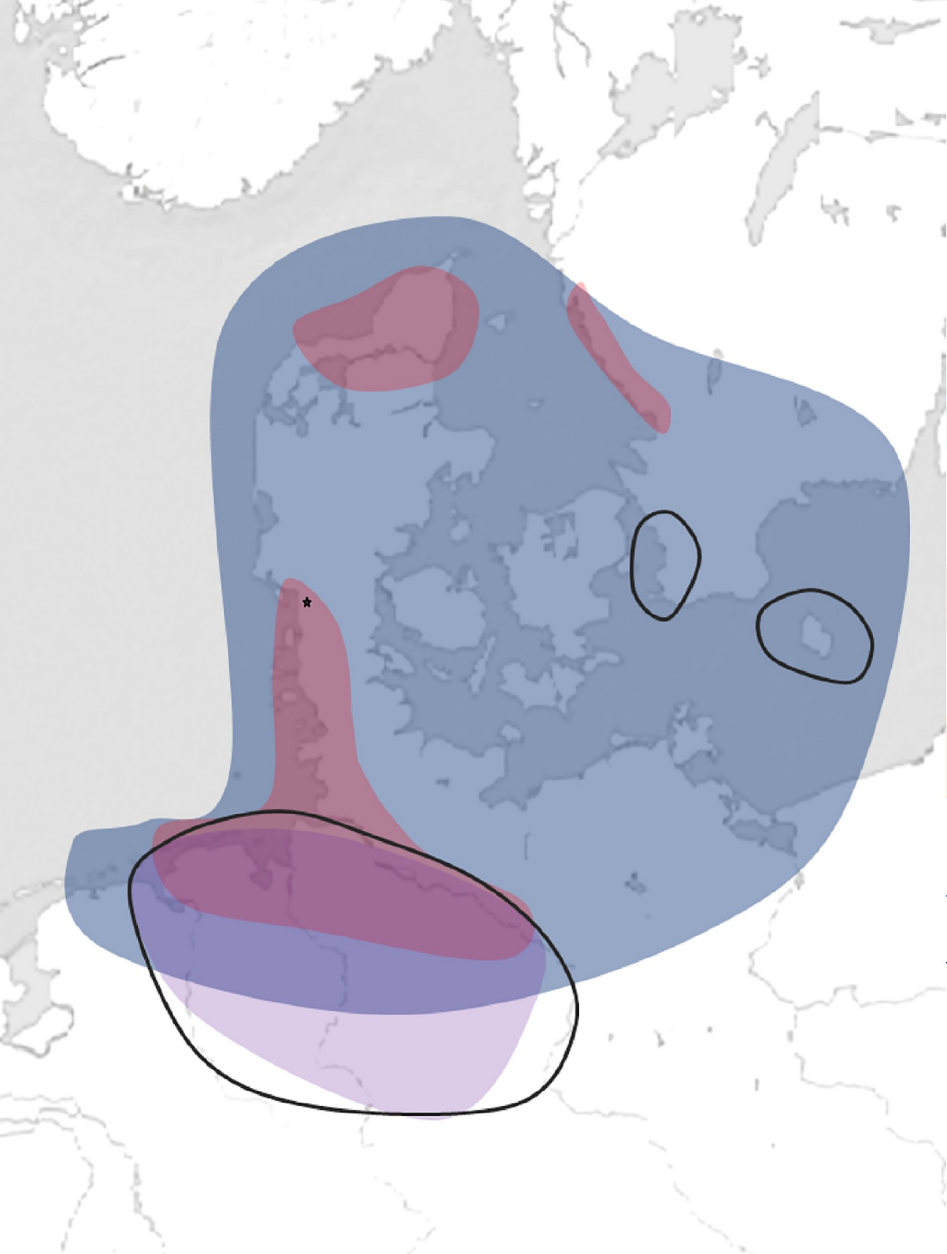
Distribution map of regional burial practices in the 8^th^-early 9^th^ century in South Scandinavia, Eastern Frisia and Northern Germany.

Although some burial practices at Ribe’s earliest cemetery are also documented at other sites in the immediate area, several have their best parallels outside this zone (Fig 4). Cremation deposits in urns, with similar types of deposited objects, are common in Ribe’s surroundings and in the North Frisian islands of the 8^th^ century [44], but also further down the historical coast of Frisia, with a similar taste for using both wares of regional type and Frankish pottery imports [46: table 14], and even similar animal species [47]. Cremation deposits in pits are widely distributed throughout Southern Scandinavia/Northern Central Europe [48: vol. 1, 63; 74–76], and cannot be connected to a specific region. Inhumations oriented N–S are encountered in Scania and on the island of Bornholm, whereas a combination of N–S or S–N inhumations may be found along the Frisian coast and in today’s Northern Germany [48: vol. 1, 51–57]. Boat graves with complete logboats and parts of larger boats are documented in Southwest Jutland from the Late Roman Iron Age to the Viking Age [49]. They are also widely distributed throughout the North Sea region, and were particularly common in Western Scandinavia in the Viking Age [48: vol. 1, 95–99; 50]. In both Frisia and Saxony, W–E inhumations, with or without coffins, became increasingly common over the course of the 8^th^ century, probably as a result of the Christian influence brought by the Frankish expansion [46: table 13, 15; 51: 318–20; 372–9], although these remained rare in Southwest Jutland until the 9^th^ century [44: 260–262]. Horses and dogs are also found in both regions [52], with very close parallels along the river Elbe, with respect to the harness and saddle of the Ribe horse grave [53]. Artefactual evidence from the Ribe graves points in the same direction [54].

The burial customs at Ribe’s earliest cemetery present discrete regional distributions beyond Ribe’s immediate area, and differ from the pattern otherwise known from the rest of Jutland in this period. The map shows the regional distribution of some of the most characteristic types: cremation deposit on the surface and/or in pit (blue); cremation deposit in urn (red); inhumation, oriented W-E and S-N (purple); horse and dog, as special deposit or animal burial (black polyline). Besides Ribe, all these types are only represented in the northern Elbe-Weser region and along the Eastern Frisian coast. Ribe is indicated by a black star. Graphics: Sarah Croix, drawing based on data published in [48: vol. 1, 39–113]. Background map retrieved from Natural Earth. Free vector and raster map data @ naturalearthdata.com. Reproduced under the copyright terms of use of Natural Earth.

The abovementioned results should be viewed with caution, owing to current limitations in the available comparative data set. Burial practices in Southwest Jutland are well-documented for the Roman and Early Germanic Iron Age, but from c. 550, burials almost disappear from the archaeological record until the 9^th^ century, possibly because they consisted mostly of cremations deposited on the surface of the ground. This applies to the rest of the Jutland peninsula and Funen [55]. In contrast, burial practices are well-documented for the 8^th^ century in the Frisian and Saxon regions. It should also be emphasized that, on many levels, the burial practices documented in Southwest Jutland are very similar to those of the Northern Frisian coast [44: 298–300]; hence stressing the strong cultural ties between the two regions. Nevertheless, several burial practices in Ribe have their strongest parallels in the Eastern and Northern Frisian areas, and in the Elbe region, and no exact parallel in either the immediate area, that is, Southwest Jutland, or in Jutland as a whole. Therefore, from an archaeological, contextual perspective, Ribe’s earliest cemetery reflects burial practices that are both regional, meaning those of Southwest Jutland, and supra-regional, in a much broader geographical sense.

As previous debates have shown, the interpretation of burial practices as indicators of cultural affinity is far from straightforward. Mortuary rituals have been shown to provide a context for negotiating identities and social relations, as well as for an idealized representation of the dead [e.g. 56, 57]. The role of deposited objects in this process has been underlined [58], and the use of dress accessories as markers of ethnicity criticized [26, 27]. However, other aspects of the mortuary ritual, such as the form of the burial, the treatment of the corpse, the use of grave markers, and their combination, may be seen as reflecting the communities of specific regions, rather than ethnic groups [59]. By focusing on these aspects, the analyses of burial practices presented here suggest that a large part of the buried population at Ribe’s earliest cemetery may have had, if not actual foreign origins, at least cultural affinities—projected or adopted by the mourners and/or the dead—from regions outside Southwest Jutland.

## Strontium isotope analyses

Strontium isotope analyses provide a means of examining human geographic mobility and the provenance of archaeological human remains at the individual level [28, 29, 60–62]. Strontium has four naturally occurring isotopes (^88^Sr, ^87^Sr, ^86^Sr and ^84^Sr), and the ratio of ^87^Sr/^86^Sr is used as a geological tracing system for provenance [63]. The ^87^Sr/^86^Sr ratio is dependent on the age of the rock and its composition. Strontium enters the body through food and water, and is absorbed by human tissue (e.g. bone and teeth), where it replaces calcium [28, 29]. In particular, the strontium isotope ratio of tooth enamel in humans is of interest, since the enamel mineralizes during childhood (with the exception of the third molar), and does not remodel after its formation. Importantly, it is also generally resistant to contamination (diagenesis) [29 and references therein].

To investigate the geographical provenance of individuals by applying the strontium isotope system, the baseline or isoscape ranges of the isotopic composition of local bioavailable strontium must be known. Most of Denmark (with the exception of the island of Bornholm) consists of a pre-Quaternary geological basement composed primarily of Tertiary and Cretaceous sediments. These are overlaid by glaciogenic sediments deposited during the Ice Ages. Ribe is located west of the Maximum glacial advance line (~22.000 B.P.), which delineates the maximum expansion of the ice cover in Jutland during the last Ice Age -the Weichsel Ice Age. West of this line, the landscape is characterized by Weichselian outwash plains and Saalian glacial deposits, which form shallow hills rich in coarse sand and clay soil, and between them are meltwater valleys with fine sand. Detrital carbonates (with relatively high strontium concentrations) also occur in sediments of the West Jutland glaciogenic province, in some areas constituting up to ~50% of the clastic components in moraine sediments [64].

Several studies conducted during the last decade aimed to shed light on the question of which type of proxy (e.g. plants, water, fauna, soils, etc.) is suitable for establishing baselines that may be used as reference maps for provenance studies [e.g. 65, 66]. Despite these efforts, there is still no consensus as to which type of proxy is the most suitable for delineating the isotopic range of bioavailable strontium signatures of an area [66]. Furthermore, there are ongoing debates about potential anthropogenic factors, such as the use of fertilizers [67] and agricultural lime [68], which aim to investigate the effects of such additives on the biosphere. However, a very recent study of a soil profile and the respective pore waters from farmland in the glaciogenic outwash plain of Western Jutland shows that strontium from agricultural lime is effectively retained near the surface [69]. Hence, agricultural liming does not appear to contaminate the groundwater-supported surface waters in Western Jutland, making baseline maps based on such waters relevant to provenance studies [69].

In the last decade, several baseline studies have been conducted in Denmark, which aim to establish the bioavailable strontium isotope range of this region. Although some studies aim to provide a general overview based on surface water [70] and fauna samples [71], others concentrate on detailed baseline investigations in the vicinity of the archaeological site of interest (often including several types of proxies, i.e. plants, soils, water and fauna) [61]. Jointly, these efforts have yielded a baseline for present-day Denmark (including the area of Ribe in Southwest Jutland) with strontium isotope signatures that range from ^87^Sr/^86^Sr ~0.7081 to 0.7111 [e.g. 61, 70–72]. Only the island of Bornholm (located south of Sweden in the Baltic Sea) has elevated bioavailable strontium isotope signatures, owing, among other things, to its Precambrian basement, which dominates a large part of the island [73]. These ranges for present-day Denmark seem to be consistent with the recent bioavailable strontium isotope baseline mapping of > 1200 soil samples from Europe [67]. Therefore, in order to avoid confusion, when we refer to the baseline for “present-day Denmark,” we exclude the area of Bornholm, unless otherwise stated.

It is important to state that the strontium isotope system can identify geographic mobility only in cases where the bioavailable strontium possesses distinctive source-area ranges. In the present study, the cultural affinities of Ribe’s community may be understood as reaching beyond Southwest Jutland, to the coastal regions of the Wadden Sea, including the Frisian coast of Northern Germany and the Netherlands. These areas have strontium isotope baselines that partially overlap those of present-day Denmark, making it impossible to distinguish geographic mobility between these regions based solely on the strontium isotope system [65, 74–77].

### Sampling strategy and methodology

In order to identify provenance and possible geographic mobility through childhood, we collected multiple samples from each individual [e.g. 61]. We conducted 43 strontium isotope analyses of a total of 21 individuals, that is, all individuals whose preserved material was suitable for analysis. We sampled the first, second, and third molars whenever possible. In cases where no teeth were suitable for analyses, tissue from the petrous bone (pars petrosa) was sampled instead of first molars. In a few cases, we sampled both the petrous bone and first molar to investigate affinity, as previous studies have shown that petrous bones are good substitutes for first molars, and are not altered by cremation processes [78]. Additionally, we measured a surface water sample from the River Ribe, to complement the previously-mentioned baseline for present-day Denmark [e.g. 61; 70; 72]. For the baseline and human remains measurements mentioned above, we followed the procedures described in previous studies [61; 73; 78].

Tooth enamel and petrous bone samples (a few mg) were taken with a Dremel tool (diamond burrs) after surface pre-cleaning with a diamond sanding bit to remove potential contamination or/and dentine. The samples were dissolved in a 1:1 mixture of 30% HNO_3_ (Seastar) and 30% H_2_O_2_ (Seastar). The samples typically decomposed within 15 minutes, after which the solutions were dried on a hotplate at 80°C. Samples were taken up in a few drops of 3 M HNO_3_ and loaded on disposable extraction columns with a 0.2 ml stem volume. The columns were charged with 200 μl intensively pre-cleaned mesh 50–100 SrSpec™ (TrisKem Int.) resin. The elution recipe essentially followed that used by Horowitz et al. [79], scaled to our needs. Strontium was eluted/stripped by pure deionized water, and then the eluate was dried on a hotplate. All samples were prepared at the Danish Center for Isotope Geology, Department of Geosciences and Natural Resource Management, University of Copenhagen.

Samples were dissolved in 2.5 μl of a Ta_2_O_5_–H_3_PO_4_–HF activator solution, and directly loaded onto previously outgassed 99.98% single rhenium filaments. Samples were measured at 1,200–1,300°C in dynamic multi-collection mode on a VG Sector 54 IT mass spectrometer equipped with eight faraday detectors (Department of Geosciences and Natural Resource Management, University of Copenhagen). Five-nanogram loads of the NBS 987 Sr standard gave ^87^Sr/^86^Sr = 0.710236±0.000010 (n = 10, 2σ). The correction for inter-laboratory bias of the strontium isotope ratios of the samples studied here, for example to ^87^Sr/^86^Sr value for NBS 987 of 0.710248 [80], was not applied. Results and errors are reported in Table 2.

**Table 2:** Strontium isotope results of individuals unearthed at Ribe's earliest cemetery.

Lab. Nr.	Excavation ID	Grave ID	Main burial type	Burial subtype	Grave goods	Orientation	Phase	Sex	Age at death	Sample description	^87^Sr/^86^Sr	2SE(abs.)
KF 983	SJM 348	K141 (x61)	Cremation	Deposit in pit	X		I	Ind.	5–6	M2	0,709938	0,000011
KF 984-a	ASR 1000	G4/A191 (x60)	Inhumation	Coffin		WNW-ESE	II	Ind.	25–30	M1	0,709964	0,000011
KF 984-b	ASR 1000	G4/A191 (x60)	Inhumation	Coffin		WNW-ESE	II	Ind.	25–30	M2	0,710000	0,000005
KF 985-a	ASR 1000	G3/A25 (x14)	Inhumation	Earth-grave		SW-NE	II	Pos. F	30–35	M1	0,710255	0,000006
KF 985-b	ASR 1000	G3/A25 (x14)	Inhumation	Earth-grave		SW-NE	II	Pos. F	30–35	M2	0,710601	0,000006
KF 985-c	ASR 1000	G3/A25 (x14)	Inhumation	Earth-grave		SW-NE	II	Pos. F	30–35	M3	0,710985	0,000004
KF 986	ASR 1000	G5/A120 (x61)	Inhumation	Earth-grave		WSW-ENE	II	Pos. M	30–35	M2	0,710258	0,000004
KF 987-a	ASR 1000	G7/A121 (x64)	Inhumation	Earth-grave		WSW-ENE	II	Ind.	11–15	M1	0,710448	0,000004
KF 987-b	ASR 1000	G7/A121 (x64)	Inhumation	Earth-grave		WSW-ENE	II	Ind.	11–15	M2	0,710298	0,000006
KF 987-c	ASR 1000	G7/A121 (x64)	Inhumation	Earth-grave		WSW-ENE	II	Ind.	11–15	M3	0,710386	0,000006
KF 988-a	ASR 1000	G13/A127 (x63)	Inhumation	Earth-grave		W-E	II	Pos. M	30–35	M1	0,710219	0,000005
KF 988-b	ASR 1000	G13/A127 (x63)	Inhumation	Earth-grave		W-E	II	Pos. M	30–35	M2	0,710485	0,000004
KF 988-c	ASR 1000	G13/A127 (x63)	Inhumation	Earth-grave		W-E	II	Pos. M	30–35	M3	0,710553	0,000006
KF 988-d	ASR 1000	G13/A127 (x63)	Inhumation	Earth-grave		W-E	II	Pos. M	30–35	pars petrosa	0,710013	0,000006
KF 989-a	ASR 1000	G14/A123 (x50)	Inhumation	Earth-grave		W-E	II	Ind.	10–12	M1	0,709875	0,000005
KF 989-b	ASR 1000	G14/A123 (x50)	Inhumation	Earth-grave		W-E	II	Ind.	10–12	M2	0,709927	0,000005
KF 989-c	ASR 1000	G14/A123 (x50)	Inhumation	Earth-grave		W-E	II	Ind.	10–12	M3	0,710015	0,000006
KF 990	ASR 1000	G15/A30 (x33)	Inhumation	Earth-grave		W-E	II	Ind.	Adultus	pars petrosa	0,710086	0,000006
KF 991-a	ASR 1000	G16/A35 (x11)	Inhumation	Earth-grave		W-E	II	F	30–40	M1	0,709566	0,000004
KF 991-b	ASR 1000	G16/A35 (x11)	Inhumation	Earth-grave		W-E	II	F	30–40	M2	0,709531	0,000004
KF 991	ASR 1000	G16/A35 (x11)	Inhumation	Earth-grave		W-E	II	F	30–40	pars petrosa	0,709881	0,000007
KF 992	ASR 8	G9/A36 (x61)	Cremation	Deposit in urn	X		I	Ind.	12–19	pars petrosa	0,710420	0,000005
KF 993	ASR 8	G9/A36 (x62)	Cremation	Deposit in urn	X		I	Ind.	Infans II	pars petrosa	0,710386	0,000005
KF 994	ASR 8	G12/A1-A143 (x119)	Cremation	Deposit in urn	X		I	Pos. M	Adultus	pars petrosa	0,709907	0,000005
KF 995	ASR 8	G16/A346 (x248)	Cremation	Deposit in pit	X		I	Ind.	Adultus	pars petrosa	0,710510	0,000006
KF 996	ASR 8	G22/A463 (x370)	Cremation	Deposit in pit			I	Ind.	>12	pars petrosa	0,709505	0,000004
KF 997	ASR 8	G2/A13 (x11)	Cremation	Deposit in pit	X		I	Pos. M	40–60	pars petrosa	0,710345	0,000004
KF 998-a	SJM 129	G1/A14 (x1)	Inhumation	Earth-grave		W-E	I	Ind.	Juvenis	M1	0,713431	0,000008
KF 998-b	SJM 129	G1/A14 (x1)	Inhumation	Earth-grave		W-E	I	Ind.	Juvenis	M2	0,715025	0,000006
KF 998-c	SJM 129	G1/A14 (x1)	Inhumation	Earth-grave		W-E	I	Ind.	Juvenis	M3	0,715454	0,000011
KF 999-a	SJM 348	K242 (x580)	Cremation	Deposit in urn	X		I	Ind.	<2	M1	0,710201	0,000005
KF 999-b	SJM 348	K242 (x580)	Cremation	Deposit in urn	X		I	Ind.	<2	pars petrosa	0,709783	0,000006
KF 1000-a	SJM 348	K304 (x562)	Inhumation	Earth-grave		SE-NW	I	Pos. M	15–20	M1	0,710514	0,000004
KF 1000-b	SJM 348	K304 (x562)	Inhumation	Earth-grave		SE-NW	I	Pos. M	15–20	M2	0,710596	0,000006
KF 1000-c	SJM 348	K304 (x562)	Inhumation	Earth-grave		SE-NW	I	Pos. M	15–20	M3	0,710509	0,000006
KF 1000-d	SJM 348	K304 (x562)	Inhumation	Earth-grave		SE-NW	I	Pos. M	15–20	pars petrosa	0,710385	0,000004
KF 1001-a	SJM 348	K399 (x435)	Inhumation	Earth-grave		NW-SE	I	Ind.	Juvenis	M1	0,709604	0,000011
KF 1001-b	SJM 348	K399 (x435)	Inhumation	Earth-grave		NW-SE	I	Ind.	Juvenis	M2	0,709404	0,000004
KF 1001-c	SJM 348	K399 (x435)	Inhumation	Earth-grave		NW-SE	I	Ind.	Juvenis	M3	0,709522	0,000005
KF 1001-d	SJM 348	K399 (x435)	Inhumation	Earth-grave		NW-SE	I	Ind.	Juvenis	pars petrosa	0,709726	0,000009
KF 1002	SJM 348	K588 (x702)	Cremation	Deposit in pit	X		I	Ind.	30–50	pars petrosa	0,709958	0,000004
KF 1004-a	ASR 8	G10/A129 (x196)	Inhumation			S-N	I	Ind.	10–11	M1	0,715005	0,000013
KF 1004-b	ASR 8	G10/A129 (x196)	Inhumation			S-N	I	Ind.	10–11	M2	0,715708	0,000009
KF 860		River Ribe								water	0,708759	0,000015

**Excavation ID**: Archival journal number (ASR: Antikvarisk Samling Ribe; SJM: Sydvestjyske Museer); **Grave ID**: (G…) as published in Feveile (ed.) 2006; Feature number (A… or K…); Find number (x…). **Main type of burial**: Simplified description focusing on the treatment of the body. **Burial subtype**: Additional information on the presence/absence of container. **Grave goods**: an X indicates the presence of objects deposited in the grave. **Orientation**: Orientation of the grave expressed in cardinal points; the first point indicates the orientation of the head, when known. **Phase**: Many graves could be dated with a reasonable degree of certainty to the 8th and early 9th centuries (phase I). Only a few graves from phase II could be dated, and several graves are dated to this phase because of the similarity of burial customs and spatial concentration with dated graves. I: 8th–early 9th centuries; II: (9th–)10th century. **Sex, and age at death**: Based on osteological analysis only. There was a high degree of uncertainty for sex estimation, therefore most identifications are presented with a question mark. Age at death is also typically expressed in broad age categories. The two non-local individuals identified by strontium isotope analyses are shaded grey.

### Results of the strontium isotope analyses

**Fig 5:**
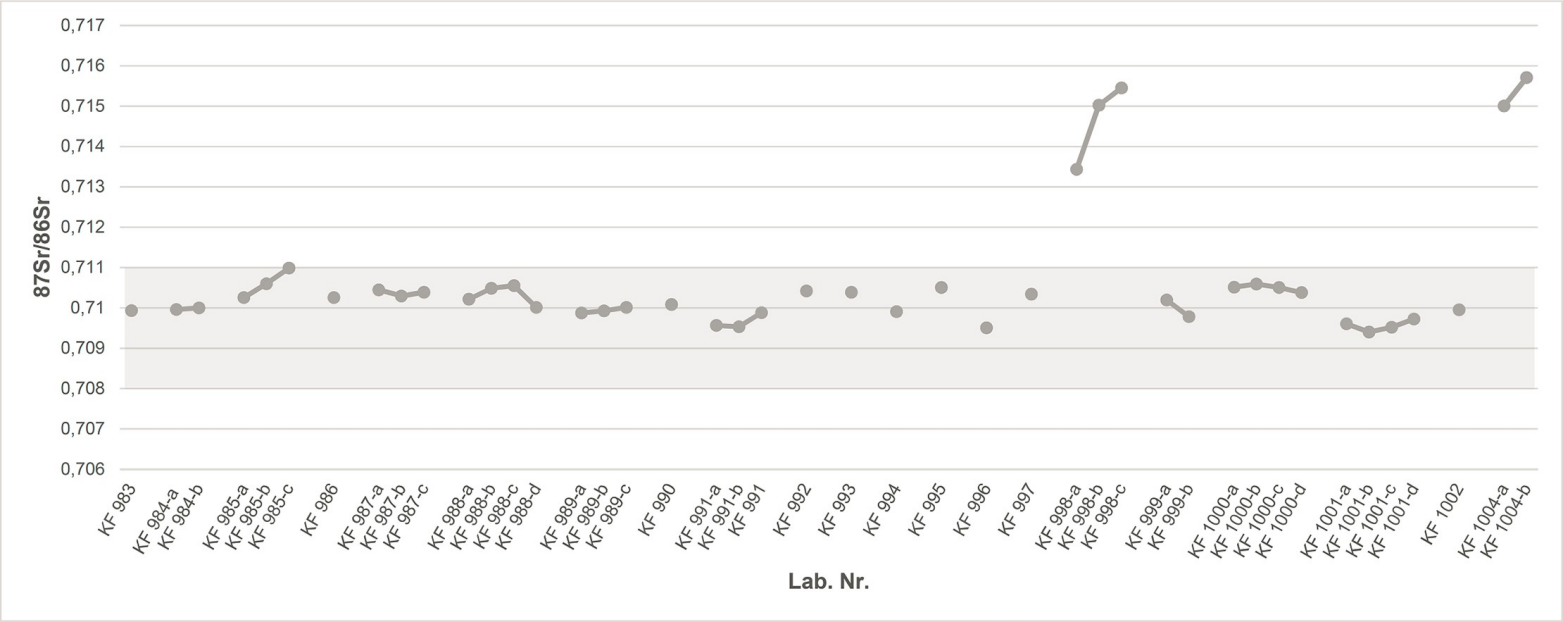
Scatter plot diagram of ^87^Sr/^86^Sr values for 21 individuals from Ribe’s earliest cemetery. The pale grey band indicates the local baseline range. Most individuals yielded strontium isotope values falling within the local baseline range. Individuals represented by several samples (e.g. M1, M2, M3 and/or pars petrosa) are depicted with linking lines. The values for the two non-local individuals, KF 998 (SJM 129 G1/A14 x1) and KF 1004 (ASR 8 G10/A129 x196), clearly differ from the bulk of the population.

The strontium isotope analyses for the 21 individuals investigated here yielded a range from ^87^Sr/^86^Sr = 0.709404 to 0.715708, and are presented in Table 2 and Fig 5. Additionally, the surface water value of the River Ribe’s water today yielded a ^87^Sr/^86^Sr = 0.708759. This value falls within the previous baseline range that seems to characterize present-day Denmark. Multiple sampling of individuals was possible in 11 of the 21 cases. The strontium isotope results reveal that most of the individuals yield strontium isotope values that fall in a relatively narrow range, between ^87^Sr/^86^Sr = 0.7094 to 0.711. Only two individuals yielded values > ^87^Sr/^86^Sr = 0.711. This suggests that most of these individuals were of “local” origin. However, as mentioned above, there are other areas outside present-day Denmark with similar or partially overlapping strontium isotope baseline ranges. For example, at present it seems impossible to distinguish between individuals from Southwest Jutland and from the Frisian coast. Based on the strontium isotope values from our dataset, only the two individuals, with values above ^87^Sr/^86^Sr = 0.711, suggest non-local origins. The two non-local individuals are KF 998 (SJM 129 G1/A14 x1) and KF 1004 (ASR 8 G10/A129 x196), with values that range from ^87^Sr/^86^Sr ~0.713 (KF 998) to ^87^Sr/^86^Sr ~0.716 (KF 1004).

The multiple sampling strategy seems in this case to provide additional information on the two non-local individuals. Earlier studies have suggested that significant differences in the strontium isotope ratios (in the order of around the fourth and third digit) between paired teeth of the same individual might indicate movement [81]. In their study, Slater, Hedman and Emerson investigated multiple molars of a number of individuals from the key site of Cahokia in present-day USA (dating to the 11^th^ to 14^th^ centuries). They reported two individuals (#41, 42) with strontium isotope differences of >0.0010. The authors wrote that “Significantly, for both of these individuals, the later-developing tooth (one M2 and one M3) has a local ratio while their early-developing tooth has a non-local ratio, suggesting these individuals came to the American Bottom during later childhood or adolescence. Differences of this magnitude are less easily attributed to local post-weaning dietary changes alone and, we suggest, reflect movement between areas with distinctly different ^87^Sr/^86^Sr signatures during the period of tooth formation” [81]. In our study, individual KF 998 yielded strontium isotope results with a relatively large (and greater than the one mentioned above) difference between the sampled teeth from ^87^Sr/^86^Sr ~0.713 in the first molar to ^87^Sr/^86^Sr ~0.715 in the second and third molars. Whereas there can be different explanations to why we observe this large difference, we suggest that this individual may have moved during childhood, from an area with strontium isotope values of ^87^Sr/^86^Sr ~0.713 to an area with more radiogenic values. Alternatively, the area in which this individual lived may have been characterized by a broad baseline range that included both values. If one assumes that relocation took place, it seems to have happened early in childhood, as we can see from the difference between M1 and M2. M1 normally starts to mineralize in utero, and finishes doing so at c. 3 years of age, whereas M2 mineralizes later (from c. 2 to c. 8 years of age) [82]; hence, this potential relocation or mobility might have happened during the timespan in between the enamel mineralization of these two teeth. In contrast, individual KF 1004 yielded similar strontium isotope values in both M1 and M2, indicating that this individual probably lived in the same area throughout early childhood, and moved to Ribe only later.

Of the 19 individuals with local signatures, 9 were also analyzed using the multiple sampling strategy. Although in some cases the strontium isotope ratios of individuals in the “local” group differ relative to one another, most of the 9 individuals for which multiple sampling was possible seems to have relatively similar strontium isotope values during early life. This may be interpreted in several ways. One possible scenario is that these individuals did not change their area of residence during childhood, as the values are consistent throughout the period of growth. Alternatively, if they did move, they did so between areas with similar strontium isotope baseline ranges, and therefore the strontium isotope composition in their tissues did not change significantly.

## Discussion

Ribe’s emporium is located at the crossroads of the North Sea and the Scandinavian spheres, and plays a key role in the study of early medieval urbanism. From the 8^th^ century onwards, Ribe’s trade networks included Nordic areas such as Norway and the Baltic Sea, and more distant regions, such as the Mediterranean and the Middle East [34, 35], with the bulk of the contacts being with the lower North Sea region and the Rhine river valley [36, 37]. The burial practices represented by Ribe’s earliest cemetery also testify to cultural affinities that reached beyond Jutland’s hinterland, and stress Ribe’s involvement in supra-regional social networks. On the other hand, the results of our strontium isotope analyses show that most of these individuals yielded values that fall within the local baseline range, which might suggest a local origin in Jutland or Ribe itself. Yet, the fact that the local baseline range also partly overlaps with that of the coastal regions of the Wadden Sea, including the Frisian coast of Northern Germany and the Netherlands, maintains the possibility open that many individuals at Ribe’s cemetery did originate from these closely connected, but distinct regions from Southwest Jutland. Still two individuals have strontium isotope ratios that clearly differ from the majority of the individuals studied herein, pointing to other areas of origin [65, 67, 73, 83–88].

From the methodological point of view, the correlation among the four individuals (KF985, KF988, KF1000 and KF 1001) for which we conducted strontium isotope analyses of both M1 and pars petrosa indicates that there is a strong correspondence between these two types of tissue. Other recent studies also reinforce the validity of petrous bone as an alternative sample tissue to of M1 [89, 90].

**Fig 6:**
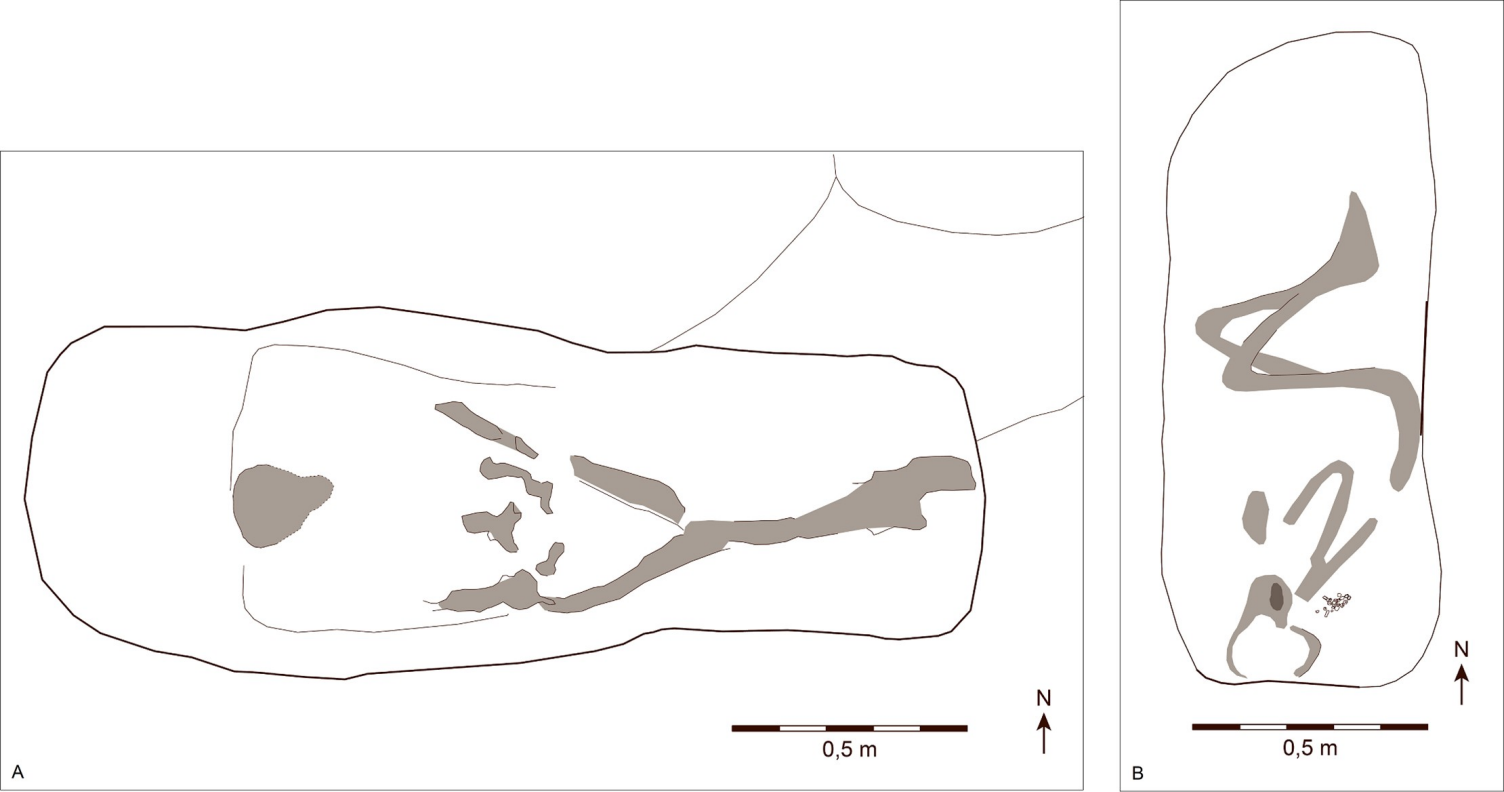
Two non-local individuals from Ribe’s earliest cemetery. Plan of the graves of the two non-local individuals identified by strontium isotope analyses: (A) SJM 129 G1/A14 and (B) ASR 8 G10/A129. Skeletal remains were poorly preserved, and the light grey areas indicate where bone material could be excavated and documented. SJM 129 G1/A14 was inhumed without grave goods, whereas ASR 8 G10/A129 was accompanied by a small assemblage of beads. Their concentration behind the head of the body suggests that they ornamented a headdress. Graphics: Museum of Southwest Jutland and Graphics Department, Moesgård Museum.

One of the two individuals who we interpret as being of non-local origin, KF 998 (SJM 129 G1/A14), is an inhumation, oriented W–E with the head to the W, the body in a supine position, with no objects deposited along their side (Fig 6A). The burial did not show traces of a coffin [91: 10–11]. The study of the dental remains suggested that this individual was a juvenile. The grave is undated, but is presumably from the early phase of the cemetery. It is tempting to see a Christian influence on its burial custom. Inhumation, oriented W-E with the head to the W, the body in supine position and without grave goods alongside them, are characteristics commonly associated to a Christian burial tradition in an early medieval context. In the 8^th^-early 9^th^ century, it was performed mostly in the Frankish areas to the south, and increasingly in Frisia and Saxony [51].

The other non-local individual, KF 1004 (ASR 8 G10/A129) is also an inhumation; however, it differs in that the deceased was oriented S–N with the head to the S (Fig 6B). The body was placed in a crouched position facing E, and laid on a layer of organic material, perhaps a textile or animal skin. A small concentration of glass beads at the back of the head may be interpreted as a necklace or an ornamented headdress. The study of the dental remains suggests that this individual was a juvenile. The grave finds date the grave to c. 760/770–800 AD, based on bead-type chronology [92: 73–74]. The headdress, including the type of glass beads, has a close parallel at the cemetery of Rullstorf, in the Elbe region [93: 61–63, abb. 16, color plate B]. In this cultural context, a set of beads deposited in the grave is typically interpreted as indicating a female individual, which may thus also be the case for the Ribe find. The S–N orientation of the inhumation at Rullstorf, with the head to the S, also strengthens this connection.

Considering the currently available baseline knowledge of northern and central Europe, combined with the archaeological context, it seems that the non-local KF998 individual may have originated in the Rhine area of central and southern Germany [e.g. 65, 67, 83, 84, 88], whereas the non-local KF 1004 individual seems more difficult to assess. Indeed, the geobiochemical and the archaeological interpretations seem to point in different directions, suggesting that the cultural environment in which the individual was born may not have been the same as that of the people who buried them. However, these two individuals may also have originated from other areas not mentioned here, but with similar baselines ranges, for example Norway [e.g. 67].

Regardless of the difficulty of pinpointing possible areas of origin of these individuals, some similarities emerge. Both individuals were sub-adults, inhumed during phase I of the cemetery, and both show similar radiogenic values in their second molars, which may indicate that they spent parts of their childhoods in similar geographical areas. However, there are also significant differences in the way they were treated after death, suggesting that even if they lived in similar regions at some points in their childhoods, the people who buried them had rather different ideas about how this should be done.

In the following section we discuss some interesting features of the group of individuals (19 in total) whose strontium isotope ratios fall within the local baseline range of present-day Denmark. Our data set does not suggest any obvious differences with respect to the various phases of the cemetery, that is, between 8^th^/early 9^th^-century graves and presumed 10^th^-century graves. However, in several cases, the burial treatments have close archaeological parallels outside the region of Ribe. The grave of individual KF 994, whose cremated remains were placed in an urn together with horse-harness fittings, in the first half of the 8^th^ century, may be compared to finds in the local area (only 15km north of Ribe, at St. Darum) [94], as well as with finds in Eastern Frisia, in present-day Northern Germany [e.g. 95]. The same applies to a baby, KF 999, whose cremated remains were placed, at some point during the 8^th^ century, inside a pitcher imported from the region of Mayen near Koblenz [96]. At this point in time, cremation burial would not have been conducted in the eastern part of the Christian Frankish kingdoms, where the pitcher originated. However, the re-use of Rhenish vessels as urns is well-attested along the Frisian coast, up to the North Frisian Islands [46: table 14]. Also, individual KF1000 was inhumed crouched and oriented N–S with the head to the S, a practice that would have been rare in the surroundings of Ribe and Jutland in the 8^th^–early 9^th^ centuries, but which is better documented in the Elbe region during this period [48: vol. 1, 51–57].

Although possible geographic mobility cannot be excluded, because the strontium isotope baseline ranges of the abovementioned areas partially overlap [e.g. 67], in these particular cases it is difficult to assess the question of geographic mobility based on this methodology. Despite the geographical distance, contacts between coastal Frisia and Southwest Jutland, including Ribe, would have been facilitated by sailing along the shores of the Wadden Sea. Consequently, it is not possible to determine whether actual geographic mobility and/or intense and frequent contact is the cause of the strong cultural affinities with communities established along the Frisian coastline, displayed by several individuals buried at Ribe’s earliest cemetery.

The relatively small variation among the strontium isotope values between most of the local individuals, also in the cases that multiple sampling was possible, does not indicate a community visibly characterized by geographic mobility between childhood and adulthood. A single adult individual, KF985, possibly a female, shows some differences between the strontium isotope composition of her M1, M2 and M3, which may indicate that during childhood, she moved among areas that have strontium isotope baseline ranges that fall within the local baseline range for present-day Denmark. The manner of her burial, a simple inhumation grave without a coffin or deposited objects, oriented SW–NE, dated to phase II of the cemetery, is not diagnostic of any regional cultural affinity in Northwest Europe in this period. Although one of the non-local individuals was also tentatively identified as female, based on the artefacts deposited alongside them in the grave, the data set is too small, and the sex estimation too uncertain to interpret female individuals as particularly geographically mobile. However, the cases of sub-adult geographic mobility identified here are thought-provoking, as they suggest that the (adult) agents who are commonly assumed to have traveled along the routes of the exchange networks were at times accompanied by young individuals.

Ribe’s results are difficult to contextualize in the light of the current state of research, as to this date there is only a limited number of published studies of urban mobility in an early Viking-Age context based on the results of strontium isotope analyses. The emporium Ribe is typically compared to sites such as Birka, Hedeby, Kaupang, and Reric / Gross Strömkendorf, where large cemeteries contemporary with the trading sites have been excavated. In Birka, 42 human samples have been analyzed so far applying the strontium isotope methodology, revealing both local and non-local individuals [14]. However, it represents only a very small portion of the more than 1100 graves documented at the site. At Reric / Gross Strömkendorf, in present-day Germany (near the Baltic Sea), strontium isotopic values were measured for three individuals yielding ratios of ^87^Sr/^86^Sr = 0.70892, 0.709102, and 0.709013, which were interpreted as local [48: vol. 2, 297]. However, as mentioned earlier, these values fall also within the baseline range of the region along the North Sea coastline. In addition, strontium and genomic variation identified among the buried population at Sigtuna (present-day Sweden) has been interpreted as reflecting the presence of a large number of non-local individuals [97]. Sigtuna was founded more than two centuries after Ribe, in a markedly different social, cultural, political and economic context, which prevents a direct comparison. Further comparative studies with other early medieval urban sites in Northwestern Europe are needed in order to get a better understanding of this phenomenon in its historical context.

## Conclusion

Although mortuary practices are often interpreted to be indicators of the (cultural) affinities of the person buried—based on burial practices, grave goods, and their association to specific regions—the geographic origin of individuals may be investigated through geobiochemical analyses, such as strontium isotope analyses. Today, a combination of both approaches is widely used in archaeological investigations.

In the context of maritime trade networks in early medieval Northwestern Europe, the flow of materials and things suggests the existence of connecting communities from various regions. Although human geographic mobility is considered to be a prerequisite for these exchanges, the social dynamics characterizing the emporia through which these networks were articulated, and the extent to which they hosted mobile and/or more permanently settled communities still needs further scrutiny. The results of our analysis of the burial remains from Ribe’s earliest cemetery reveal that most of the sampled individuals have strontium isotope values that fall within the previously characterized baseline range of present-day Denmark, including Southwest Jutland, where Ribe is located. On the other hand, the diversity of burial practices reflects cultural affinities that may be connected to communities outside present-day Denmark, for example, with the Frisian coast (stretching all the way from the Netherlands to Denmark) and the northern part of the river Elbe region (Germany). These areas have strontium isotope baselines that partially overlap those of Jutland (Denmark), thus limiting the possibility of identifying geographic mobility of individuals who traveled between them, when using this approach. Hence, based on the combination of the contextual archaeology and strontium isotope results, we cannot exclude the possibility that some of Ribe’s “local” individuals originated from other regions with strontium isotope baselines that overlap that of present-day Denmark. Only two of 21 individuals had strontium isotope values that did not fall within the local baseline range, and therefore are regarded as originating from an area other than, and possibly distant from, Ribe. Both these individuals were sub-adults, and were inhumed in the oldest phase of the cemetery while the emporium was flourishing in the 8^th^ and 9^th^ centuries.

Viewed in light of our current knowledge of Viking-Age Ribe, our results seem somewhat surprising. Although the material culture unearthed in the dwellings and workshops of the emporium suggests the presence of, or at least contact with, individuals belonging to communities from local, regional, and distant areas, our study has not revealed geographic mobility from distant regions on a large scale. The apparent scarcity of “long-distance” travelers, originating from regions outside present-day Denmark and the lower North Sea region, may be because the number of individuals analyzed here represents only a fraction of Ribe’s Viking-Age community. Our results do not exclude the possibility that more non-locals settled and died in Ribe. Similarly, our approach does not provide final conclusions on the geographic mobility of the adult actors in trade. “Local” individuals may have travelled extensively during adulthood, both in areas with similar strontium isotope baseline ranges as present-day Denmark, and in other areas, without it appearing in our data. Our current results suggest that, if Ribe’s emporium did attract a permanent relocation of individuals who were not born there, it may have done so for individuals originating in communities in the Wadden Sea and Elbe regions, which show a high degree of cultural integration with Southwest Jutland. Further research may contribute to enlightening us about the role of regional actors, beyond the local area, in Ribe’s social networks, and thus, in the early stages of the urbanization of Scandinavia.
